# Child-Focused Mental Health Interventions for Disasters Recovery: A Rapid Review of Experiences to Inform Return-to-School Strategies After COVID-19

**DOI:** 10.3389/fpsyt.2021.713407

**Published:** 2021-10-05

**Authors:** Gabriela Gómez, Armando Basagoitia, María Soledad Burrone, Marlene Rivas, María Teresa Solís-Soto, Sean Dy Juanco, Hugh Alley

**Affiliations:** ^1^Institute of Educational Sciences, Universidad de O'Higgins, Rancagua, Chile; ^2^Institute of Health Sciences, Universidad de O'Higgins, Rancagua, Chile; ^3^School of Public Health, Touro University California, Vallejo, CA, United States

**Keywords:** COVID-19, children, mental health, interventions, crisis and disaster

## Abstract

There is a worldwide need for mental health interventions to address the mental health needs of children under 12 who are returning to school in the post-COVID-19 environment. The basic characteristics of child-focused, post-crisis interventions are currently unknown, but they are essential for developing high-quality, expedient RTC programs. We conducted a rapid systematic review, via established PICO methodology, to appraise the characteristics of such interventions. We queried databases (PubMed, PsycInfo, ERIC) for English and Spanish publications describing mental health interventions to reduce mental health symptoms and sequelae among children exposed to disasters and other community crises. We described the following characteristics: type of intervention, length, number of sessions, number of staff delivering the intervention, and other characteristics. A total of 18 original articles met the inclusion criteria: 11 correspond to a controlled trial type of study and 15 addressed PTSD after disaster or crisis situations. Cognitive-behavioral therapy (CBT) was the most common intervention type, school-based/related interventions were the most common method, and five articles described an important role of teachers as mediators of therapy.

## Introduction

The COVID-19 pandemic has fundamentally changed the way we live since December 2019. Our day-to-day activities vary widely due to uncertainty about novel forms of SARS-CoV2, local spikes in COVID-19 incidence, local governmental restrictions, and the inevitable fear, stress, and panic that follow disruption. Countries faced the pandemic with a variety of public health policies, some of which lead to new and stressful situations due to job losses and economic insecurity. Besides that, the tragic loss of loved ones and the impact of quarantines separating and disrupting traditional support systems have imposed mental health burdens on the general population and profoundly affected vulnerable groups.

The vulnerability of children to anxiety and depression has been described in both the early (1) and subsequent (2, 3) stages of the epidemic (4, 5). This suggests that investigating program design and strategies to mitigate the impact on childhood development is a legitimate priority among public policy discussions.

In this sense, and mindful of the best practices available to plan the return to schools, we performed a systematic search for mental health interventions in children returning to school after health crises and epidemics prior to COVID-19. Preliminary searches found few successful evidence-based interventions to use as a starting point for further strategies in this specific target group and context.

Therefore, we developed a rapid review protocol to search for information on mental health interventions for children exposed to community crises or disasters, describe the results, and identify a range of options that may assist government, academic, and community leaders concerned for children and the effects of the pandemic on our next generation.

## Methods

We applied the practical guide for rapid reviews to strengthen health policy (6) from WHO to conduct our review and report on the evidence regarding mental health interventions among children post-crisis. We applied the PROSPERO (7) guidance notes to register the review and report the findings of this review. Finally, to minimize duplication of efforts, we searched for registered reviews that overlapped with the focus of this study, looking for articles describing the characteristics of post-crisis return-to-school interventions focused on children.

### Search Strategy

A comprehensive search strategy was developed by the research team, and was used to identify articles in three electronic widely used databases: Medline; APA PsycInfo (Proquest); and the Education Resources Information Center (ERIC) (for search details, see Supplementary Table 1). Since this was a rapid review that aimed to be systematic and expedient; we did not include gray literature in our search strategy.

The time range and language parameters were: 2,000 to present and English and Spanish. The searches used database-specific subject headings and keywords in natural language.

To capture the most recent publications, database searches were run on 14 September, 2020, 14 October, 2020, 14 December, 2020, and 22 May, 2021.

The search keywords were mapped to congruent MeSH and Emtree terms in different combinations, linked using the Boolean operators “AND,” “OR.” The specific keywords included: “child,” “mental health,” “well-being,” “psychosocial,” “depress,” “anxiety,” “PTSD,” “posttraumatic stress,” “grief,” “insomnia,” “disaster,” “Intervention,” “therapy,” “treat,” and “support.”

### Selection Criteria

All primary research studies were identified and evaluated using PICO (Population, Intervention, Comparison, Outcome) (8), and included the following:

- Population: Studies examining children (age 14 or younger) exposed to crisis or catastrophe situations who received mental health interventions.
- Intervention: Studies reporting any type of mental health interventions that occurred after a crisis or catastrophic situation (natural disasters, human-made disasters). A mental health intervention was defined as any interpersonal or informational activities, techniques, or strategies that target biological, behavioral, cognitive, emotional, interpersonal, social, or environmental factors with the aim of improving health functioning and well-being.
- Studies were excluded if they did not focus on mental health interventions, or if they did not describe intervention results.
- Comparison: Different mental health intervention strategies or no intervention at all.
- Outcomes: Studies reporting the setting, development, and effectiveness of mental health interventions designed to improve health functioning among children returning to school after crisis situations.
- Types of studies: To objectively determine the intervention results, we included quantitative study designs (randomized controlled trials [RCTs], non-randomized controlled trials [NRCTs], and non-randomized non-controlled trials [NRNCTs]).
- Language: Studies in English and Spanish.
- Country: No limits were defined.

A total of 811 titles and abstracts were screened for relevance and possible inclusion. Two reviewers independently searched the databases, screened studies for inclusion (in two steps: first by titles and abstract, and later by full-text scanning and reviewing), and extracted the data.

**Figure 1:**
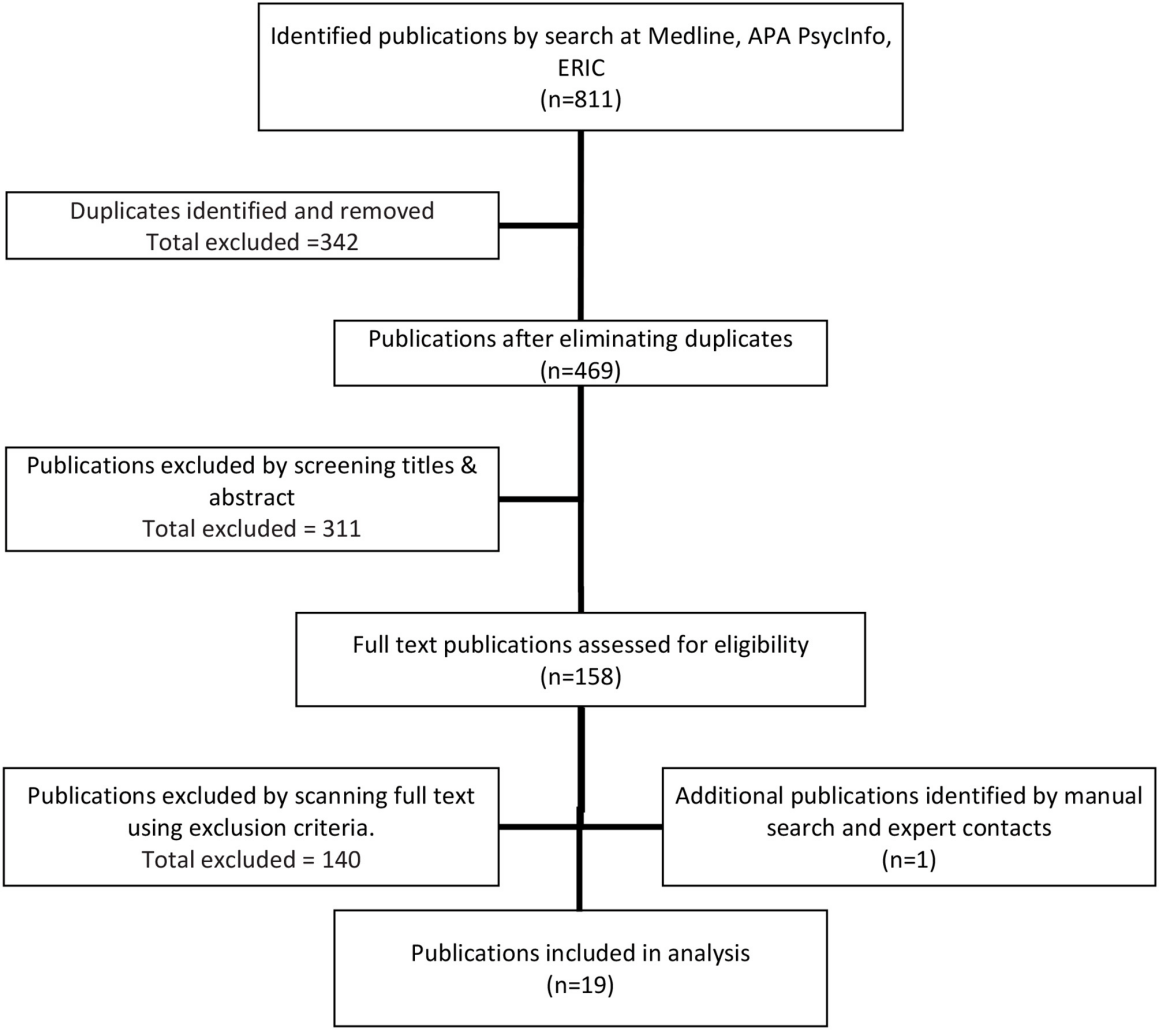
Flow diagram of study selection.

The differences in results were discussed and a consensus was reached on the final findings presented in this review. An independent group of mental health professionals and methodologists were on the team to advise on the differences of findings between the two reviewers. Through this process, 18 primary research studies and 21 systematic reviews were selected for inclusion. The number of articles identified at each stage of the selection process is listed in Figure 1.

### Data Extraction

We extracted basic information about study characteristics such as location, year, study type, publication, and sample size. We also collected data about mental health problems, context characteristics (crisis-related intervention), and mental health intervention characteristics such as psychological intervention type, intervention length, number of sessions, setting, delivery characteristics (e.g., Face to face vs. distance delivery, and individual vs. group), delivery subjects, and changes of symptom occurrence. We developed a data extraction form a priori to gather the mentioned information.

No risk-of-bias assessment was performed since the objective of the study was to explore basic information on the topic, which is an option in developing rapid systemic reviews.

## Results

We organized results by the following topics: Study & Crisis characteristics and Intervention characteristics.

### Characteristics of the Included Studies

There were 18 primary research studies were selected for this research. These studies were conducted in 11 countries: Chile, Denmark, Haiti, Israel, Netherlands, New Zealand, India, Indonesia, Italy, Canada, and the USA. There were 12 studies related to natural disasters including earthquakes, hurricanes, tsunamis, and fires. There were also 6 studies related to man-made disasters including war, terrorism, and explosions. There was 1 study was related to the recent COVID-19 pandemic.

There were 6 studies with an RCT design, 6 studies with an NRCT design, and 7 studies with an NRNC design. Sample sizes ranged from 32 (Chembot et al.) to 1,684 (Wolmer et al.). There were 16 studies that assessed PTSD symptoms and only that three assessed other specific mental health issues. Table 1 presents these studies (author, publication year, title, study design, and sample size) as well as crisis characteristics (type of crisis, location) and which mental health problems were addressed.

**Table 1:** Characteristics of the included studies.

**Author**	**Year**	**Title**	**Study design**	**Sample Size**	**Type of crisis**	**Crisis location**	**Mental health problem addressed**
Chemtob, Nakashima, Hamada	2002	Psychologial intervention for postdisaster trauma symptoms in elementary school chidlren	NRCT	248	Hurricane	USA, Kauai	PTSD symptoms
Chemtob, Nakashima, Carlson	2002	Brief treatment for elementary school children with disaster-related posttraumatic stress disorder: a field study	RCT	32	Hurricane	USA, Hawaii	PTSD symptoms
Vijayakumar, Kannan, Kumar, Devarajan	2006	Do all children need intervention after exposure to tsunami?	NRCT	230	Tsunami	India, Srinivasapuram	PTSD symptoms
Salloum, Overstreet	2008	Evaluation of individual and group grief and trauma interventions for children post disaster	RCT	56	Hurricane	USA, Louisiana	PTSD symptoms, depression, traumatic grief, distress
CATS Consortium	2010	Implementation of CBT for youth affected by the World Trade Center disaster: matching need to treatment intensity and reducing trauma symptoms	NRCT	306	Terrorist attack	USA, New York	Trauma symptoms
Wolmer, Hamiel, Laor	2011	Preventing children's posttraumatic stress after disaster with teacher-based intervention: a controlled study	NRCT	1,488	War	Israel (Southern/Gaza Strip)	PTSD
Wolmer, Hamiel, Barchas, Laor	2011	Teacher-Delivered Resilience-Focused Intervention in Schools With Traumatized Children Following the Second Lebanon War	NRCT	983	War	Israel	PTSD, fear, stress
de Roos, Greenwald, Hollander-Gijsman	2011	A randomized comparison of cognitive behavioral therapy (CBT) and eye movement desensitization and reprocessing (EMDR) in disaster-exposed children	RCT	52	Explosion (fireworks factory)	Netherlands, Enschede	PTSD
Jaycox, Cohen	2011	Children's Mental Health Care following Hurricane Katrina: A Field Trial of Trauma-Focused Psychotherapies	RCT	118	Hurricane	USA	PTSD
Rønholt, Karsberg, Elklit	2013	Preliminary Evidence for a Classroom Based Psychosocial Intervention for Disaster Exposed Children with Posttraumatic Stress Symptomatology	NRT, NCT	108	Fire	Denmark	PTSD
Wolmer, Hamiel, Slone, Faians	2013	Post-traumatic reaction of Israeli Jewish and Arab children exposed to rocket attacks before and after teacher-delivered intervention	NRT, NCT	1,684	War	Israel (Northern)	PTSD symptoms
Blanc, Bui, Mouchenik, Derivois	2014	Prevalence of post-traumatic stress disorder and depression in two groups of children one year after the January 2010 earthquake in Haiti	NRCT	58	Earthquake	Haiti, Port-au-Prince	PTSD symptoms, depression
Garfin, Silver,	2014	Children's Reactions to the 2010 Chilean Earthquake: The Role of Trauma Exposure, Family Context, and School-Based Mental Health Programming	NRT, NCT	117	Earthquake	Chile	PTSD
Stasiak, Merry, Frampton, Moor	2016	Delivering solid treatments on shaky ground: Feasibility study of an online therapy for child anxiety in the aftermath of a natural disaster	NRT, NCT	42	Earthquake	New Zealand, Christchurch	Anxiety
Rebecca A. Graham,	2017	School based post disaster mental health services: decreased trauma symptoms in youth with multiple traumas	NRT, NCT	112	Hurricane	USA	Trauma symptom
Dawson, Joscelyne, Meijer	2017	A controlled trial of trauma-focused therapy versus problem-solving in Islamic children affected by civil conflict and disaster in Aceh, Indonesia	RCT	64	War	Indonesia, Aceh	PTSD, depression and anger symptoms
Trentini, Lauriola,	2018	Dealing With the Aftermath of Mass Disasters: A Field Study on the Application of EMDR Integrative Group Treatment Protocol With Child Survivors of the 2016 Italy Earthquakes	NRT, NCT	701	Earthquake	Italy, Umbria	PTSD, distress, anxiety, depression, anger and need for help
Moor, Williman, Drummond	2019	‘E' therapy in the community: Examination of the uptake and effectiveness of BRAVE (a self-help computer program for anxiety in children and adolescents) in primary care	NRT, NCT	1,026	Earthquake	New Zealand, Cantebury	Anxiety
Malboeuf Hurtubisea, Léger-Goodes	2021	Philosophy for children and mindfulness during COVID-19: Results from a randomized cluster trial and impact on mental health in elementary school students	RCT	37	COVID-19 pandemic	Canada, Quebec	Anxiety, inattention

*RCTs, randomized controlled trials; NRCTs, non-randomized controlled trials; NRNCTs, non-randomized non-controlled trials; PTSD, Post-traumatic stress disorder*.

### Intervention Characteristics

The characteristics of the interventions are presented in Supplementary Table 1, including therapeutic intervention, setting and duration of the intervention, number of sessions, face/distance delivery, group/individual delivery, party responsible for mediating the intervention, procedures' description level, effectiveness reported, effectivity assessment measuring tool, intervention effectivity, and effect size.

Therapeutic methods include both well-known and novel interventions. A total of 10 studies described cognitive-behavioral therapy (CBT) as the main therapy delivered (9–12) during the intervention and CBT variations including trauma-focused CBT (13–16) and computerized CBT BRAVE-ONLINE (17, 18), two studies described the use of eye movement desensitization and reprocessing (EMDR) (19, 20), one study referred to use of stress inoculation training (SIT) (21), one study described philosophy for children (P4C) and mindfulness-based interventions, one study described the use of a national program in Chile: “Skills for Life” (22), and four other studies did not specify the therapy or program, but described the contents of the interventions used (23–26).

School-based/related interventions were the most common, with 12 studies that reported using this approach, three studies that examined interventions related to clinical settings, and four that were performed at the participant's home or in the community.

There were three studies that described sessions with a duration of 1 week, one study that described sessions with a duration of 5 weeks, four reported up to a month, four studies reported up to 6 months, and seven studies that did not report this data. There were eight studies that reported up to five sessions, four studies up to 10, three studies up to 14 sessions, and only three studies that did not report this data.

There were 15 studies concerning face-to-face interventions and four studies on interventions via online spaces. There were 10 studies that reported individual interventions, five studies that reported group sessions, three studies that reported using both strategies, and only one study that did not report the strategy used.

Mediators of the intervention had variable levels of training. Only five interventions described an important role of teachers as mediators of therapies, and the rest (14) describe clinicians, therapists, or other professionals as leading the interventions.

### Effectiveness of the Interventions

All studies except one (Blanc et al.) reported successful results and interventions' effectivity. The most frequent tool used to measure changes in mental health symptoms was the UCLAS PTSD Reaction index, which was mentioned in five studies. In general, the tools used were varied in number and characteristics.

Finally, the description of the effect size varied greatly among different studies, partially due to the different study designs, assessment tools used and statistical strategies selected. A summary of the outcome measurement based on the main mental health outcomes of each study is presented in Supplementary Table 1.

## Discussion

As a new educational season has begun, schools around the world will face many challenges in areas such as biosafety, vaccination, blended learning, and other topics. Nevertheless, managing the mental health impact of the COVID-19 pandemic on the entire population, and especially on vulnerable populations such as children, is an important pillar for return-to-school strategies.

While previous return-to-school strategies and mental health interventions after health-related crises focused on children have not been widely described in scientific literature, experiences describing mental health interventions with children after crises or catastrophic situations are an important information source to inform stakeholders and help develop realistic plans to take care of children's health. Thus, the primary research articles described in this review provide insight into previous experiences that could help orient new strategies for dealing with COVID-19 mental health burdens among children.

This review found that, given the previous experiences described, there is a highly relevant opportunity to improve health interventions by involving different actors from the educational community to develop interdisciplinary and participative strategies. As some articles described, the participation of parents and teachers is relevant since they could become agents who can improve the efficacy of the interventions and also their efficiency. The training of teachers to implement interventions could increase the system capacity to reach and follow the children over a long time period. However, to properly train teachers for this role, coordinated planning and resource allocation strategies should be developed to assure these results.

We did not perform the risk bias assessment analysis due to time constraints and the search for the information scope of the objectives, and although this is not uncommon in rapid reviews, we consider it a limitation that could be corrected in future research. Nevertheless, we are confident that the information presented provides an insight into the studies' characteristics and their reported findings, to provide guidance about the different options and characteristics of successful mental health interventions with children after crisis situations.

While there were some successful interventions that implemented distance delivery strategies via computers and thus could become an attractive option to develop new interventions, we consider that there should exist cultural contextualized strategies to avoid losing patients in the long term following the intervention.

While we did not actively search for an efficacy difference between psychological interventions types, it was clear that CBT was the most common intervention, and given that is a widespread strategy we consider this to be reasonable. Nevertheless, we think there is a need to increase evidence about which mental health interventions (or combinations of interventions) with children after crises have better results.

Also, we would like to stress the high variety of tools used in different studies to assess mental health symptoms changes before and after interventions. We do consider that a common strategy is difficult to use in different contexts, but the use of different tools also represents challenges to compare effectivity among mental health interventions. Therefore, we consider that more research is needed to clarify the differences between health interventions and assessment tools.

## Author Contributions

AB, GG, MR, MSB, MS-S, and SD: conceptualization, methodology, and formal analysis. AB, GG, MR, MSB, MS-S, and HA: writing—reviewing and editing. GG and MSB: project administration and supervision. All authors contributed to the article and approved the submitted version.

## Funding

This research was developed as part of the project URO-1895 which received funding from the Education Ministry in Chile.

## Conflict of Interest

The authors declare that the research was conducted in the absence of any commercial or financial relationships that could be construed as a potential conflict of interest.

## Publisher's Note

All claims expressed in this article are solely those of the authors and do not necessarily represent those of their affiliated organizations, or those of the publisher, the editors and the reviewers. Any product that may be evaluated in this article, or claim that may be made by its manufacturer, is not guaranteed or endorsed by the publisher.
