# Supplementary material for: Child-Focused Mental Health Interventions for Disasters Recovery: A Rapid Review of Experiences to Inform Return-to-School Strategies After COVID-19
Source: Front Psychiatry. 2021 Oct 5;12:713407. doi: 10.3389/fpsyt.2021.713407 (PMC8524184; doi:10.3389/fpsyt.2021.713407)
Supplement: Supplementary file 1 [file Table_1.docx]

**Supplementary Table 1.**

| **Author** | **Therapeutic interventions** | **Setting** | **Duration** | **Number of sessions** | **Delivery 1: Face to face or Distance** | **Delivery 2: grupal or Individual** | **Intervention mediator** | **Effectivity assessment instruments** | **Intervention effectivity** | **Effect size** |
| --- | --- | --- | --- | --- | --- | --- | --- | --- | --- | --- |
| Chemtob, Nakashima, Hamada | Not specified | School | 1 week | 4 | Face to face | Individual / Grupal | Therapists | TESTS Child Reaction Index (CRI); The Children’s Depression Inventory (CDI); The Revised Children’s Manifest Anxiety Scale (RCMAS) | MH symptoms improved | The CRI treated wave had significantly lower scores (mean score 11.65) than the untreated wave (mean score 20.32); t34=2.76; P=0.01 |
| Chemtob, Nakashima, Carlson | EMDR | School | 1 week | 4 | Face to face | Individual | Therapists | Test Kauai Recovery Inventory (KRI); Child PTSD Reaction Index (CRI) | MH symptoms improved, reduced health visits, Perception of the Helpfulness of Treatment | CRI: Group 1 decreased by 54.93% and Group 2 decreased 42.93% at posttreatment. Contrasts on the means yielded a significant pretreatment versus posttreatment difference, F(1,30)=37.35, p <.0009, d=1.55 RCMAS: F(1,30)=15.30, p< .0009, d=0.78 CDI: F(1,30)= 18.05, p< .0009, d=.69 |
| Vijayakumar, Kannan, Kumar, Devarajan | CBT Cognitive behavioral treatment | Community | 24 weeks | 6 | Distance | Individual | Psychologists and volunteers | Test Youth Self Rating form - (YSR); Child PTSD Reaction Index (CPTSD-RI) | Hyperactivity problems reduced | Hyperactivity problems: (Z = 2.41, p = 0.016) Anxiety problems: (Z = 1.556, p = 0.120) Affective problems: (Z= 0.723, p = 0.470) Somatic problems: (Z=1.783, p = 0.075) Oppositional problems: (Z= 0.596, p=0.551) Conduct problems: (Z=0.049, p=0.961) PTSD related symptoms: (Z=0.045, p=0.964) |
| Salloum, Overstreet | Individual: CBT + narrative exposure therapy | After-school program | 10 weeks | Not specified | Face to face | Both | Clinicians | The Mood and Feelings Questionnaire–Child Version (MFQ–C), Treatment satisfaction | MH symptoms improved without difference between group or individual intervention | Difference in PTSS between pre and posttreatment: t(48) = 7.81, p = .001, d = 1.16 Difference in depression symptoms: t(48) = 3.49, p = .001, d = .53 Difference in traumatic grief scores: t(31) = 4.13, p = .001, d = .73 |
| CATS Consortium | Trauma-specific CBT | Not specified | Not specified | 4 | Face to face | Individual | Therapists | Test PTSD Reaction Index | Trauma symptoms decreased | Difference in trauma-specific CBT mean: 36.61 (baseline) to 21.73 (after 6 months) Δ in brief CBT skill mean: 20.75 (baseline) to 12.82 (after 6 months) |
| Wolmer, Hamiel, Laor | Stress Inoculation Training (SIT) | School | 14 weeks | 14 | Face to face | Group | Teachers, school counselors | Test UCLAPTSD Reaction Index, Stress/Mood Scale | Symptoms of post-trauma and stress/mood decreased | UCLA pre-test (EMDR:31.4, CBT30.5) and post-test (EMDR:16.1, CBT:16.9) |
| Wolmer, Hamiel, Barchas, Laor | Coping enhancement | School | Not specified | 13 | Face to face | Group | Teachers | Test derived from the Child PTSD Reaction Index | improvements in children’s stress/mood and post-trauma | CBITS Baseline (PTSD: 22.0, Depression: 13.4); 10 months (PTSD: 15.8, Depression: 9.7) - TF-CBT Baseline (PTSD: 22.9, Depression: 15.4); 10 months (PTSD: 12.0, Depression: 11.1) |
| de Roos, Greenwald, Hollander-Gijsman | Exposure-based CBT | Clinical setting | 4 weeks | 4 | Face to face | Individual | Therapists | Test UCLA PTSD Reaction Index, Child Report of Post-traumatic Symptoms (CROPS) | Reduction all MH symptoms | PTSD, anxiety, and depression reduction by both treatments (p<0.001) Mean EMDR child sessions = 3.17 vs mean CBT child sessions = 4.0 |
| Jaycox, Cohen | Trauma-Focused Cognitive-Behavioral Therapy | School | 16 weeks | 16 | Face to face | Individual vs Group | Therapists, assessors, and intake workers | Test UCLA PTSD Reaction Index, Children’s Depression, Strengths and Difficulties Questionnaire (SDQ) | MH symptoms improved | PTSD total symptoms at follow-up: Intrevention (Mean 13.90, SD 11.80) and control (Mean 19.86, SD 12.91) |
| Rønholt, Karsberg, Elklit | Cognitive & narrative methods includes TF-CBT | School | 5 weeks | 5 | Face to face | Individual | N/A | Cartoon (Darryl) visual and auditory clues about PSTD | MH symptoms improved | Statistically significant reduction from Pre-treatment to Post-treatment T-test: (re-experiencing: t(94) = 4.12, p\.001; avoidance: t(96) =2.50, p\.02) and in the total number of PTSD symptoms (symptom total: t(94) = 3.76, p\.001) |
| Wolmer, Hamiel, Slone, Faians | Teacher-delivered intervention | School | Not specified | 14 | Face to face | Group | Teachers | Test related to stress and mood & questions from the CPTSD Reaction Index | MH symptoms improved | Stress/mood: F(1, 712) = 7.62, p < .01, η2 =0.02 PTSD: F(1, 661) = 155.56, p < .001, η2 = 0.33 Children's functioning: F(1, 311) = 0.36, p > .05 |
| Blanc, Bui, Mouchenik, Derivois | Psychosocial support (remedial workshop, creative & play activities) | Clinical setting | Not specified | Not specified | Face to face | Not specified | Counselors | Test Child Post-Traumatic Stress-Reaction Index(CPTS-RI), The children's depression inventory(CDI), The child behavior check-list CBC | Without effectiveness | CPTS-RI: t=1.28, p=0.20 PDI-C: t=-31, p=0.75 CDI: t= 1.55, p=0.12 |
| Garfin, Silver, | SFL specific therapy | School | Not specified | 10 | Face to face | Group | Psychologists, social workers, educators | Test Teacher Observation of Classroom Behavior Revised for Chile TOCA-RR), Pediatric Symptom Checklist for Chile (PSC-CL), child UCLA PTSD | MH symptoms improved | Mean worry for children assigned to indicated prevention activities was 1.58 (SD 0.98) and for children assigned to universal prevention activities only was 2.06 (SD 1.24); this difference was statistically significant t(115) 2.07, |
| Stasiak, Merry, Frampton, Moor | Computerised CBT (BRAVE-ONLINE): | Participant's home | Not specified | 6 to 10 | Distance | Individual | BRAVE-ONLINE trainers (2 clinical psychologists, 1 occupational therapist). | Test Anxiety Disorders Interview Schedule for DSM-IV: Child and Parent versions (ADIS-C/P), Spence Children’s Anxiety Scale (SCAS), Mood & Feelings Questionnaire (MFQ-S) Short version, Child Health Utility 9D (CHU9D), Children’s Global Assessment of Functioning (CGAS) | MH symptoms improved, quality of life | ADIS CP symptom decrease: z = −3.46, p = .001 SCAS total 38.22 to 15.52; p = .003 MFQ total 11.27 to 4.97; p < .001 |
| Rebecca A. Graham, | CBT models including TF-CBT and CBITS | School | 1 week | 5 | Face to face | Individual | Clinicians | "UCLA PTSD-RI (Part III), Children’s Depression Inventory (CDI; short form), Anger Expression Scale for Children" | MH symptoms improved with both interventions | There were significant reductions in post-traumatic stress disorder on self-reported (t(131.26) = −9.26, p < 0.001) and caregiver-reported (t(170.65) = 3.53, p = 0.001) measures and anger (t(127.66) = −7.14, p < 0.001). |
| Dawson, Joscelyne, Meijer | Trauma-focused Cognitive Behavioral Therapy (CBT) | Clinical setting | 5 weeks | Not specified | Face to face | Individual | CMH lay-counsellors, no formal mental health training | Trauma Symptom Checklist for Children (TSCC) | MH symptoms improved, reduction in overall posttraumatic stress symptoms as well as subscales assessing anxiety, anger, depression, dissociation, overt dissociation, and fantasy dissociation. | PTSD decrease pre to post: (t(76.04)=−7.43, p<0.001) PTSD decrease pretreatment to follow-up: (t(131.26)=−9.26, p<0.001)  Depression decrease pre to post: (t(62.47)=−1.07, p=0.29) Depression decrease pre to followup: (t(129.14)=−0.91, p=0.36)  Anger decrease pre to post:(t118=−4.52, p<0.001)  Anger decrease pre to follow-up: (t(171.28)=2.88, p=0.005) |
| Trentini, Lauriola, | EMDR-Integrative Group Treatment Protocol (EMDR-IGTP) | School | 3 weeks | 3 | Face to face | Group | EMDR – IGTP therapists | Emotion Thermometers, Children’s Revised Impact of Event Scale (CRIES-13) | MH symptoms improved, reducing emotional disturbances and post-traumatic symptoms | Distress time*age: F(5.604), p<0.004 Distress time*gender: F(10.572), p<0.001 Anxiety time*age: F(3.544), p<0.030 Anxiety time*gender: F(17.708), p<0.001 Depression time*gender: F(7.218), p<0.001 Anger time*age: F(9.581), p<0.001 CRIES time*time elapsed: F(17.331), p<0.001 CRIES time*age: F(72.185), p<0.001 CRIES time*gender: F(5.693), p<0.004 |
| Moor, Williman, Drummond | BRAVE_TA (therapist assisted version of BRAVE-ONLINE) | Participant's home | Not specified | 6 to 10 | Distance | Individual | BRAVE therapists - Clinical psychologists | Children's Anxiety Scale (CAS8) | MH symptoms (anxiety) improved | CAS8 mean changes by number of sessions completed: 1-2 sessions: -0.7 (-1.4 to -0.1), p < 0.001, d=0.19 3-4 sessions: −2.7 (−3.4 to −1.9), p < 0.001, d=0.70 5-7 sessions: −3.2 (−4.1 to −2.4), p < 0.001, d=0.84 8 or more: −3.6 (−4.4 to −2.9), p < 0.001, d=0.94  Number sessions completed by age:  7 years: 5.1 sessions 12 years: 4.4 sessions  17 years: 3.0 sessions |
| Malboeuf Hurtubisea, Léger-Goodes | Mindfulness-based intervention (MBI), Philosophy for children (P4C) | Teleconferencing platform | 5 weeks | 5 | Distance | Individual | Trained undergraduate students in psychology | Behavior Assessment Scale for Children-3rd edition, self-report scale (BASC III).  3 items related to anxiety, 4 items related to inattention. | P4C intervention: MH symptoms improved.  MBI intervention: MH symptoms not improved. | Significant effect of condition on mental health difficulties (F (1, 29) = 5.58, p = .016, partial η2 = 0.2).  P4C group: post-test (Mpost, adjusted for baseline = 3.67).  MBI group (Mpost, adjusted for baseline = 5.35).  P4C group: Mental Health symptoms pre to post (t(18) = 2.77, p = .01).  MBI group: Mental Health symptoms pre to post (non signiticant, *p* = .24). |

CBT: Cognitive Behavioral Therapy

CBITS: Cognitive Behavioral Intervention for Trauma in Schools

EMDR: Eye movement desensitization and reprocessing

IGTP: Integrative Group Treatment Protocol

SFL: Skills for Life

TF: Trauma-focused
